# Malignant mixed Mullerian tumour of the uterus

**DOI:** 10.3332/ecancer.2013.302

**Published:** 2013-04-04

**Authors:** S K Rajshekar, B Guruprasad, PN Shakunthala, Praveen Rathod, Uma Devi, UD Bafna

**Affiliations:** 1 Department of Gynaec-Oncology, Kidwai Memorial Institute of Oncology, DR MH Marigowda road, Bangalore-560029, India; 2 Department of Medical Oncology, Kidwai Memorial Institute of Oncology, DR MH Marigowda road, Bangalore-560029, India

**Keywords:** Malignant Mixed Mullerian Tumor (MMMT), Chemotherapy, Chemoradiotherapy

## Abstract

**Background:**

A malignant mixed Mullerian tumour (MMMT) of the uterine corpus is an extremely rare and aggressive malignancy. There are very few studies regarding the outcome of MMMT patients in India. Hence, we conducted the present study to analyse the outcome of MMMTs at our institute.

**Objective:**

To study the clinical profile, prognostic features, and treatment outcome of MMMT with multimodal therapy.

**Method:**

A five-year retrospective study of the MMMT cases diagnosed and treated at our centre was conducted. Twenty patients with pathological proven diagnosis of MMMT treated at our institute from January 2007 to May 2012 were analysed. These patients underwent comprehensive surgical staging followed by adjuvant therapy in the form of chemotherapy alone or chemoradiotherapy. These patients were analysed for event-free survival (EFS), and their outcomes were correlated with histology, therapy, myometrial invasion, and the stage of disease.

**Results:**

A majority of these patients presented with postmenopausal bleeding. Endometrial biopsy was diagnostic in only 20% of the patients. Of the 20 patients who underwent surgery, 18 patients received adjuvant therapy. At median follow-up of 16 months (range 3–30 months), the EFS was 30%. No difference in outcome was noted based on tumour histology (heterologous versus homologous). Concurrent chemoradiation improves local control and may delay recurrence but has shown little survival advantage.

**Conclusion:**

MMMT is an aggressive tumour of the uterine corpus. A negative endometrial biopsy does not rule out the diagnosis. Poor outcome is noted in patients with advanced-stage disease and myometrial invasion. The optimal adjuvant treatment for this uncommon disease is yet to be established, highlighting the need for larger multicentric studies on MMMTs.

## Introduction

A malignant mixed Mullerian tumour (MMMT), also termed uterine carcinosarcoma, is an extremely rare tumour, comprising only 1–2% of uterine neoplasms [1]. These tumours are a dedifferentiated or metaplastic form of endometrial carcinoma [2]. Based on recent data, this tumour is now considered to be uterine epithelial carcinoma rather than sarcoma [3, 4]. The management of MMMTs has seen several advances in recent decades. Despite the use of aggressive adjuvant therapy, only modest improvement in survival is noted over the last couple of decades.

Since MMMTs are extremely rare and there is a paucity of data, we conducted the present study to analyse the outcome of this type of tumour in India.

## Materials and methods

This retrospective study included MMMT patients who were diagnosed and treated at our institute between January 2007 and May 2012. Patients who were referred to our institute for recurrent disease following primary treatment at another hospital were excluded from the analysis. The case records of these patients were analysed in detail for demographic profile, clinical features, and treatment outcome. The diagnosis was established on the basis of the final histopathological examination report after surgery by World Health Organization criteria. Patients were classified according to the 2009 FIGO staging system for endometrial carcinoma. Patients with good performance status (0-2 ECOG scale) received concurrent chemoradiotherapy as adjuvant therapy, while others received only chemotherapy. Combination chemotherapy with paclitaxel (175 mg/m^2^) and carboplatin (AUC 6) was administered every three weeks. Radiotherapy included pelvic external beam radiotherapy of 45 grays in 25 fractions over five weeks followed by 30 grays low-dose rate vaginal brachytherapy via single application. Chemoradiotherapy arm patients received single-agent carboplatin during radiotherapy. The patients were followed up by clinical and radiological examination every three months after therapy.

The outcome was correlated with histology (homologous versus heterologous tumours), therapy (chemotherapy alone versus chemoradiotherapy), myometrial invasion, and the stage of disease. Progression and recurrence were taken as events. The event-free survival (EFS) was evaluated using the Kaplan–Meier curve (SPSS 16 – SPSS Inc., USA).

## Results

The clinical characteristics of the 20 patients analysed are listed in Table 1. The median age of presentation was 56 years (range 47–70 years). A majority (76%) of patients were postmenopausal. Two (10%) were nulliparous. The most common presenting symptom was vaginal bleeding, followed by lower-abdominal pain. The most common associated conditions were diabetes and hypertension. Five patients had a body mass index (BMI) over 25.

**Figure 1:**
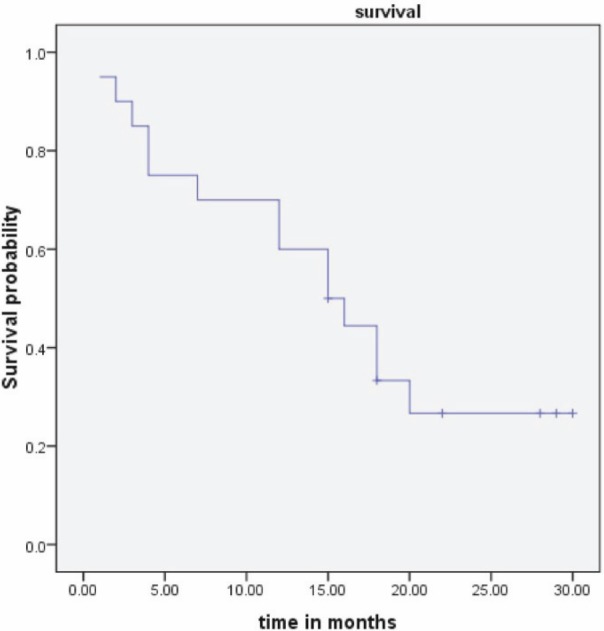
Outcome of disease in the presence of myometrial invasion

**Figure 2:**
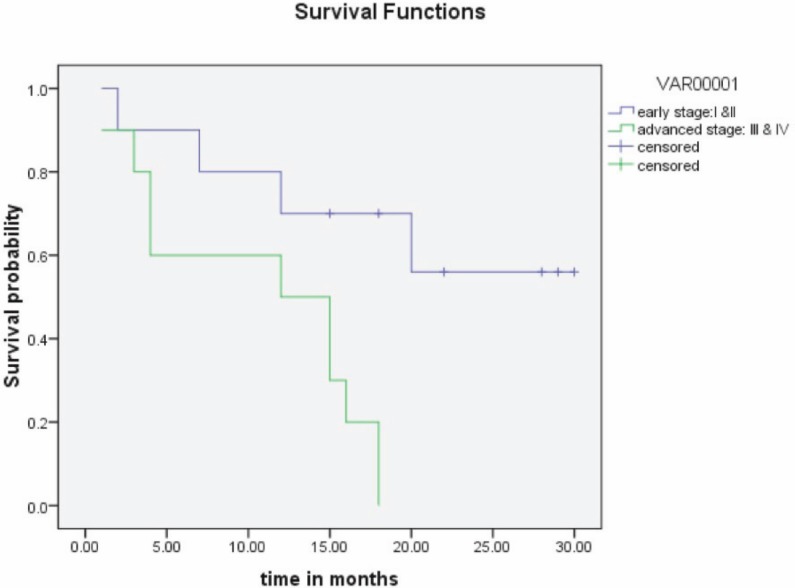
Stage of disease and outcome

Endometrial biopsy was done in all patients preoperatively. Four patients (20%) were diagnosed with MMMTs, and 12 patients (60%) were diagnosed with poorly differentiated carcinoma on endometrial biopsy. Patients commonly presented with stage III disease at diagnosis. The stage of the disease was correlated with the outcome (Figure 2). Thirteen patients (65%) had over 50% myometrial invasion (Table 2). Increasing depth of myometrial invasion was associated with a lower rate of survival, as shown in Figure 1.

Of the 14 patients (70%) having homologous tumours, 12 patients had undifferentiated sarcoma, and two had leiomyosarcoma. Of the six patients (30%) having heterologous tumours, four had rhabdomyosarcomas, one chondrosarcoma, and one osteosarcoma. The presence of heterologous stromal components did not influence the stage of the disease. The outcome of heterologous tumours was not significantly different from homologous tumours. However, our numbers were too small to compare survival between specific types of stromal components.

Among 20 patients who underwent surgery, 18 patients (90%) received adjuvant therapy. The other two patients expired within three months due to progressive disease. The median follow-up period was 16 months (range 3–30 months). Six patients (30%) received chemoradiation, and the rest of the patients received only chemotherapy as adjuvant treatment.

**Figure 3:**
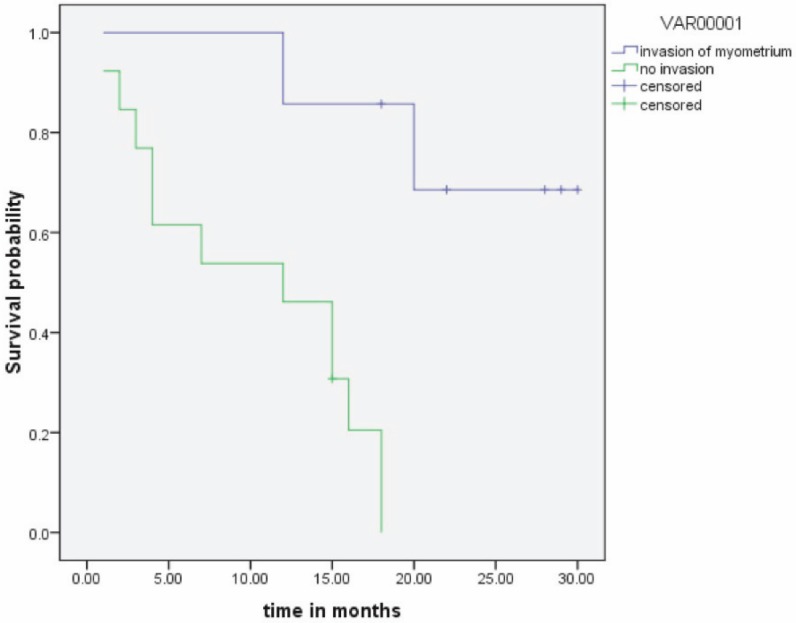
Event-free survival

The estimated two-year EFS was 30% (Figure 3). Of the six patients with chemoradiation, only two patients were disease free, and of the 12 patients in the chemotherapy arm, only 4 patients were disease free at the end of study period.

A total of 12 events occurred (25% had progressive disease, 75% had recurrence). The sites of recurrence are given in Table 3. Distant recurrence was commonly noted in patients who received chemoradiation. Of these 12 patients, seven were lost to follow-up, and the remaining five were given palliative treatment. All the patients with stage I disease were still alive at the end of the study period.

## Discussion

MMMT is the most common subtype of uterine sarcoma [5]. There is a scarcity of data in Indian context about this disease. Little is known about the clinical profile and outcome in our setting.

As observed in our study, patients with MMMTs are mainly in the postmenopausal age group, and they commonly present with vaginal bleeding. In our study, 20% of patients were obese with a BMI of more than 25; this has been reported in other studies as well [6, 7].

All of these patients underwent endometrial biopsy for diagnosis. However, only a small amount of tissue is obtained by this procedure, and a high proportion of biphasic nature of MMMTs may be missed by this method [8]. This leads to misdiagnosis and mismanagement. In our study, endometrial biopsy made the diagnosis in only four patients (20%).

Due to the aggressive nature and poor prognosis of MMMTs, various therapeutic modalities have been employed. Surgical management should include total abdominal hysterectomy and bilateral salphingo-oophorectomy, infracolicomentectomy, bilateral pelvic and para-aortic lymphadenectomy. The role of combined adjuvant radiotherapy and chemotherapy still remains to be defined. Historically, ifosfamide alone was considered the most effective drug; now, based on the GOG 161 study, ifosfamide/paclitaxel is considered the standard of care [9]. Recent data suggests that paclitaxel/carboplatin is equally effective [10] as adjuvant chemotherapy. This regimen was practiced in our study. Even with these aggressive therapies, the five-year overall survival is approximately 20–30% [8]. In our study, the estimated EFS at two years was 30%, with most recurrences occurring within one year.

Prognostic factors included the presence of myometrial invasion (p = 0.001) and advanced stage of disease (p = 0.006), which were associated with poor outcome. Other studies have reported similar data [11, 12].

Even though we noted a better outcome in patients treated with chemoradiotherapy versus chemotherapy alone, this result was not statistically significant (p = 0.9), probably due to the small number of patients in the chemoradiotherapy arm. The addition of radiotherapy also reduces the local recurrence rates. These findings of improved survival and reduced local recurrence rate have been reported by other studies as well [11, 13]. Similar to other published data [6], we did not find a statistically significant difference (p = 0.85) in survival between heterologous and homologous tumours (progression-free survival of 12 versus 13.5 months). The limitations of our study are the small sample size and short follow-up period.

## Conclusion

In conclusion, MMMTs are a rare and aggressive tumour of the uterine corpus. A negative endometrial biopsy does not rule out diagnosis. The outcome correlates with the stage of the disease and the depth of myometrial invasion. A majority of the patients with advanced-stage disease had recurrence or progression within one year. With better understanding of the disease origin, combination chemotherapy with paclitaxel and carboplatin may be beneficial. Concurrent chemoradiation improves local control and may delay recurrence but has shown little survival advantage. The optimal adjuvant treatment for this uncommon disease is yet to be established, highlighting the need for larger multicentric studies on this tumour.

## Figures and Tables

**Table 1.** Clinical characteristics

**Demographic profile**	
Median age	56 years
Postmenopausal	16 (76%)
Median BMI	24.0
**Associated condition**	
Hypertension	4 (15%)
Diabetes mellitus	8 (32%)
**Clinical features**	
Vaginal bleeding	17 (84.6%)
Lower-abdominal pain	6 (23%)
Mass per abdomen	4 (15.4%)
**Stage**	
I	6
II	4
III	8
IV	2

**Table 2.** Pathological characteristics

**Endometrial biopsy**	
Poorly differentiated carcinoma	13
Malignant mixed Mullerian tumour	4
Necrotic material	3
**Myometrial involvement**	
>50%	13
<50%	7
**Histological subtype**	
Homologous	14
Heterologous	6

**Table 3.** Site of recurrence

**Site of recurrence**		**Type of therapy**
**Local recurrence**		
Vagina	1	Chemotherapy alone
Pelvis	3	Chemotherapy alone
**Distant recurrence**		
Intra-abdominal	2	Chemotherapy alone
Lung	1	Chemoradiotherapy
Liver	1	Chemoradiotherapy

## References

[1] El-Nashar SA, Mariani A (2011). Uterine carcinosarcoma. Clin Obstet Gynecol.

[2] McCluggage WG (2002). Malignant biphasic uterine tumours: carcinosarcomas or metaplastic carcinomas?. J Clin Pathol.

[3] Kernochan LE, Gracia RL (2009). Carcinosarcoma (malignant mixed Mullerian tumor) of uterus: advances in elucidation of biologic and clinical characteristics. J Natl Compr Canc Netw.

[4] D’Angelo E, Prat J (2011). Pathology of mixed mullerian tumors. Best Pract Res Clin Obstet Gynaecol.

[5] Harlow BL, Weiss NS, Lofton S (1986). The epidemiology of sarcomas of the uterus. J Natl Cancer Inst.

[6] Ho SP, Ho TH (2002). Malignant mixed Mullerian tumours of the uterus – a ten-year experience. Singapore Med J.

[7] Song T, Choi CH, Lee YY, Kim TJ, Lee JW, Kim BG (2011). Which is worse: uterine papillary serous carcinomas or carcinosarcomas?. J Gynecol Oncol.

[8] Afonso JF (1974). Mixed mesodermal tumors of the uterus. West J Med.

[9] Homesley HD, Filiaci V, Markman M (2007). Phase III trial of ifosfamide with or without paclitaxel in advanced uterine carcinosarcoma: a gynecologic oncology group study. J Clin Oncol.

[10] Powell MA, Filiaci VL, Rose PG, Mannel RS, Hanjani P, DeGeest K (2010). Phase II evaluation of paclitaxel and carboplatin in the treatment of carcinosarcoma of the uterus: a gynecologic oncology group study. J Clin Oncol.

[11] Bosquet JS, Terstriep SA, Cliby WA (2010). The impact of multimodal therapy on survival for uterine carcinosarcoma. Gynec Oncol.

[12] Ferguson SE, Tornos C, Hummer A, Barakat RR, Soslow RA (2007). Prognostic features of surgical stage I uterine carcinosarcoma. Am J Sur Pathol.

[13] Menczer J, Levy T, Piura B (2005). A comparison between different postoperative treatment modalities of uterine sarcoma. Gynec Oncol.

